# Sensor Development for Corrosion Monitoring of Stainless Steels in H_2_SO_4_ Solutions

**DOI:** 10.3390/s21041449

**Published:** 2021-02-19

**Authors:** Miha Hren, Tadeja Kosec, Mari Lindgren, Elina Huttunen-Saarivirta, Andraž Legat

**Affiliations:** 1 Slovenian National Building and Civil Engineering Institute, Dimičeva 12, SI-1000 Ljubljana, Slovenia; miha.hren@zag.si (M.H.); andraz.legat@zag.si (A.L.); 2 Metso Outotec Research Center, P.O. Box 69, FI-28101 Pori, Finland; mari.lindgren@mogroup.com; 3 VTT Technical Research Centre of Finland Ltd., P.O. Box 1000, FI-02044 Espoo, Finland; elina.huttunen-saarivirta@vtt.fi

**Keywords:** stainless steel, hydrometallurgical industry, sulfuric acid, electrical resistance sensor, corrosion

## Abstract

Equipment made of different stainless steels is often used in the hydrometallurgical processing industry. In this study, an electrical resistance sensor was developed for monitoring corrosion in acidic solutions at high temperature. Two types of stainless steel were used as the electrode materials, namely grade 316L stainless steel (EN 1.4404) and grade 2507 duplex stainless steel (EN 1.4410). The materials and sensors were exposed to a 10% H_2_SO_4_ solution containing 5000 mg/L of NaCl at various temperatures. Results from the sensors were verified using electrochemical techniques and postexposure examination. Results showed that the microstructure played an important role in the interpretation of corrosion rates, highlighting the importance of using an appropriate stainless steel for the production of sensors. Electrochemical tests and postexposure examination both showed that the grade 2507 had a significantly lower corrosion rate compared to the grade 316L. Under industrial‑process conditions, the results for the grade 2507 sensor were promising with respect to sensor durability and performance, despite the extremely harsh operating environment.

## 1. Introduction

In the hydrometallurgical industry, the most common mechanism of failure in materials is corrosion, as the environment is characterized by elevated temperatures, high acidity and a high concentration of aggressive ions [1]. The resulting corrosion induced by such conditions can cause unplanned shutdowns, significant losses to the production of raw and end‑product materials, reduction in the energy efficiency of processes, and threaten the safety of both people and the environment [2]. As a result, reactors, tanks, pipes, mixers, filters and valves need to be designed to withstand such harsh conditions for the entirety of the equipment’s service life. Typical material choices are stainless steels containing a high percentage of expensive alloying elements which promote corrosion resistance, such as nickel and molybdenum [3,4,5]. To reduce the costs of the design, production and maintenance of the equipment, it is highly desirable to optimize both the grade of stainless steel and the amount of material used. In addition to the harsh environment, conditions during the processing of raw materials are not static. As production of the raw material progresses, the concentrations of sulfuric acid leachant and the different corrosive ions included in the raw materials constantly change [6]. In order to get an accurate material-degradation estimate throughout the entire industrial process, corrosion monitoring is necessary.

Stainless steels rely on a thin passive film formed on the steel surface to achieve their corrosion resistance. The thickness and composition of the passive film largely depend on the steel microstructure, steel composition and the media where the passive film was formed [7]. Certain environments, such as acids or aggressive halide ions, can cause the film to destabilize, which results in pit initiation or general corrosion [8]. While an adequate amount of chromium is always required to form the passive film, it is nickel and molybdenum which give stainless steels the lowered corrosion rates and pitting resistance needed during the hydrometallurgical processing of raw materials [3,5]. In certain environments, microstructural characteristics can also influence the corrosion behavior of stainless steels. Besides the different phases formed during steel production, specific dislocation structures developed during cold forming can also impact passive film stability, repassivation potential, pitting potential, and the rate at which corrosion progresses in the different forming directions [9,10,11,12]. This could have potential implications when applied to processing equipment made from steel sheets.

An electrical resistance (ER) sensor is a corrosion monitoring technique which utilizes the change in the electrical resistance of a corroding metal electrode to measure its reduction in thickness [13]. As the metal corrodes, the cross-section of the electrode decreases, causing a proportional increase in electrical resistance. ER sensors are one of the most common corrosion monitoring techniques used in different fields, including in the construction industry [14,15,16,17,18], the oil and gas industry [13,19,20], the automotive sector [21], in nuclear waste management [22,23,24] and in other, general applications [25,26,27]. Despite their popularity, however, they have not been widely applied in the hydrometallurgical industry. The general-purpose ER probes are not well suited for the extremely corrosive environments found in the hydrometallurgical industry, as the microstructure of the probe can have a large impact on corrosion rates. In addition, a major parameter influencing the measurement of electrical resistance is temperature. Since practical conditions do not allow for stable and controlled temperature variations, some form of temperature compensation in the sensor is required. This is generally achieved by using a protected reference electrode, which is exposed to the same temperature as the non-protected electrode [28]. The reference electrode can also be incorporated in the form of a Wheatstone bridge sensor design, which has temperature compensation built-in [21]. Another known downside of ER sensors is their inability to properly detect localized corrosion, meaning they are better suited to monitor systems where general corrosion is expected [15,28].

In this study, the use of an electrical resistance sensor for monitoring the corrosion of stainless steel in the hydrometallurgical industry is demonstrated. The sensor is designed to monitor the corrosion behavior of a particular grade of stainless steel, taking into account the steel microstructure, and wirelessly transfer the data measured to a remote server. Since the working electrode of the sensor is made from the same steel sheets as those used as structural material in tanks, pipes and mixers, the corrosion performance should accurately reflect the material in service. The various stages of development of the sensor are presented and evaluated: the planning and designing of the sensor, the laboratory testing of individual materials, the laboratory testing of the corrosion behavior and the industrial testing of the sensor. The results obtained using the corrosion monitoring campaigns were compared with metallurgical examination and electrochemical measurements to evaluate and verify corrosion monitoring performance. This type of corrosion monitoring can ultimately lead to better planning of service life and maintenance of the equipment and provide designers with useful information related to material selection for future equipment design.

## 2. Materials and Methods

### 2.1. The Design of the ER Sensor

**Figure 1:**
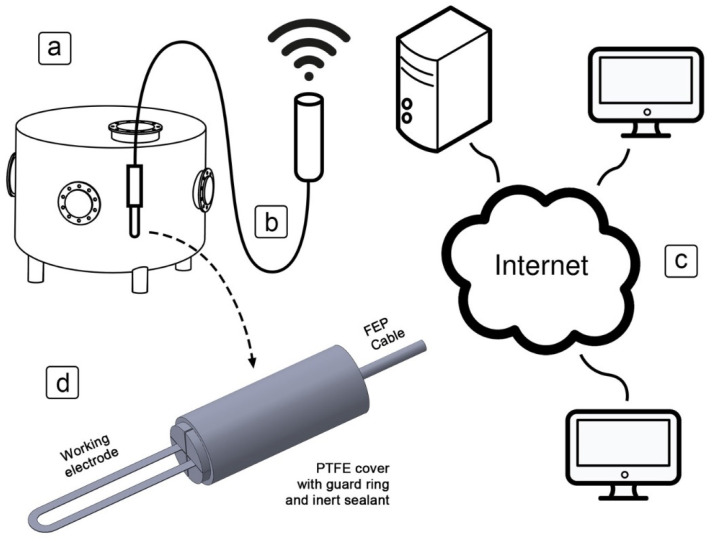
Schematic representation of the electrical resistance (ER) sensor: (**a**) the sensor inside an industrial boiler; (**b**) the datalogger connected to the sensor, with wireless transmission of the gathered data; (**c**) the server, which stores and serves data to end user devices over the internet; (**d**) the design of the sensor, comprising two polytetrafluoroethylene (PTFE) covers and a working electrode cut from a stainless steel sheet, with an inert sealant in between.

A schematic representation of the ER sensor in operation is shown in Figure 1. The exposed part of the sensor is placed inside an industrial tank (Figure 1a) and connected by wire to a datalogger (Figure 1b), which houses the measuring equipment to wirelessly transfer the recorded data to a nearby server. The datalogger consists of an A/D converter and multiple operational amplifiers which measure the electrical resistance, a microprocessor which analyses the signal and stores data locally, and an RF transmitter which communicates with the nearby server. A 15 mA electrical current is used when the electrical resistance is measured; the current was measured once every 5 min. Under regular operating conditions, the datalogger can achieve a resolution of about 10 µΩ. Initially, this represents a thickness reduction of 0.03 µm, and the resolution increases by a factor of 2 each time the resistance of the exposed electrode doubles. The maximum measurable resistance is 1 Ω (less than 10% of the original cross-section remaining).

The server is connected to the internet and provides the function of storing and serving data to end users over a web browser (Figure 1c). This allows for both graphical and tabular representation of the data in real time. The prototype of the sensor was made from a top and bottom polytetrafluoroethylene (PTFE) cover parts, and a U-shaped working electrode cut from a stainless steel sheet (Figure 1d). A copper cable made of three twisted pairs and coated with fluorinated ethylene propylene (FEP) insulation was soldered to one end of the working electrode. An inert sealant was used between the bottom and top cover, and around the cable connections, to prevent liquids from entering the protected part of the sensor.

### 2.2. Operation Principle of the ER Sensor

**Figure 2:**
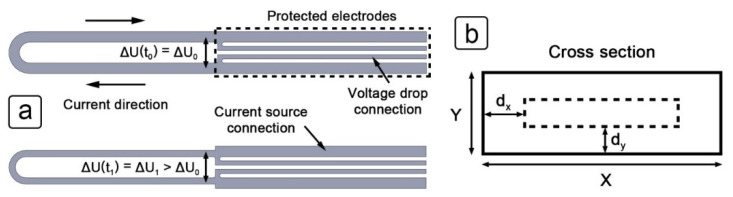
Schematic view of the principle of operation of the ER sensor: (**a**) plan view of the working electrode with labels to indicate the connections, current direction and voltage drop; (**b**) cross-section of the working electrode with the reduction in thickness illustrated in the *X* and *Y* direction.

Electrical resistance sensors measure the reduction in thickness of a metal cross-section based on the change in electrical resistance [28]. Figure 2a shows the plan view of the working electrode before (top) and after (bottom) exposure. Separate twisted pairs are used to carry the current and measure the voltage drop. An additional third twisted pair is connected to the outer electrodes to measure the voltage drop on the protected part of the sensor. These measurements compensate for both the fluctuations in current and the changes in temperature. During operation, a constant current is sent along the outer electrodes (source current), while the inner connectors are used to measure the voltage drop on the exposed part of the sensor. As the thickness of the exposed part of the sensor reduces, the measured voltage increases from ∆*U*_0_ to ∆*U*_1_.

In order to translate the measured voltage drop to changes in the thickness, certain assumptions need to be made. First, Equation (1) is used to calculate the resistances of both the exposed and the protected electrodes using Ohm’s law, while Pouillet’s law (Equation (2)) is used to connect the resistances with the dimensional properties of the electrodes.

(1) $$R_{x}=\frac{\Delta U_{x}}{I}$$

(2) $$R_{x}=\rho \frac{l_{x}}{A_{x}}$$

In order to calculate the reduced surface area of the exposed electrode (A), Equation (3) is used, which is derived from Equations (1) and (2). The assumption here is that the thickness reduction is constant along the entire electrode length. *X* and *Y* represent the original width and height of the electrode cross-section, as shown in Figure 2b. *R*_e_ and *R*_p_, respectively, represent the electrical resistance of the exposed and protected electrodes, while *R*_e,ref_ and *R*_p,ref_ represent the initial resistance of these electrodes at a reference temperature. Calculating the cross-section in this way compensates for fluctuations in the source current and temperature changes, both of which can affect the measured voltage drop.

(3) $$A=\frac{R_{e,\mathrm{ref}}}{R_{p,\mathrm{ref}}}\cdot \frac{R_{p}}{R_{e}}\cdot X\cdot Y$$

Before the new surface area can be translated into the thickness reduction of the cross-section, another assumption must be made. Figure 2b shows the reduction of the cross-section in the *X* and *Y* directions, respectively labelled *d*_x_ and *d*_y_. The rate at which the thickness is reduced during corrosion in both the *X* and *Y* directions depends on a combination of the stainless steel grade, it’s microstructure and the environmental conditions. A new constant labelled *k* is introduced, which represents the quotient of the two variables, as shown in Equation (4). This constant needs to be experimentally determined.

(4) $$k=\frac{d_{x}}{d_{y}}$$

Using this constant (*k*), the initial dimensions of the cross-section (*X* and *Y*), and the reduced surface area (*A*), *d*_y_ and *d*_x_ can be calculated using Equations (5) and (6), respectively.

(5) $$d_{y}=\frac{2\left(X+kY\right)-\sqrt{4{\left(X+kY\right)}^{2}-16k\left(X\cdot Y-A\right)}}{8k}$$

(6) $$d_{x}=k\cdot d_{y}$$

### 2.3. The Electrode Materials

Stainless steel sheets made of grades 316L (EN 1.4404) and 2507 (EN 1.4410), manufactured and supplied by Outokumpu Stainless AB, Avesta, Sweden, were chosen for sensor fabrication and the related studies. Both materials are already common in certain applications within the hydrometallurgical industry. The steel sheets were 0.5 mm thick, and either laser cutting or abrasive waterjet cutting was used to cut specimens from the steel sheets. The electrical resistivity at room temperature of grades 316L and 2507 was measured at 0.73 µΩm and 0.82 µΩm, respectively. The chemical composition of the materials is given in Table 1 and the pitting resistance equivalent number (PREN) was calculated from the alloy composition. The trade nomenclature (316L and 2507) was used throughout the research to easily identify the two steel grades.

### 2.4. Solutions for Laboratory and Industrial Validation Exposure

For the laboratory tests, a 10% sulfuric acid solution with 5000 mg/L of NaCl was prepared from analytical grade chemicals. The solution was either kept at room temperature (22 ± 2 °C) or heated to 50 ± 0.5 °C, 65 ± 0.5 °C or 90 ± 0.5 °C during testing.

The industrial validation tests were done in a solution with a variable concentration of sulfuric acid, chloride ions, fluoride ions, ferric ions and arsenic ions at a temperature of approximately 35 °C.

### 2.5. Metallographic Investigations

Stainless steel sheet specimens of grades 316L and 2507 were metallographically investigated to study their microstructure, the effects of the cutting procedures and the type of corrosion attack after exposure to the acidic solution. The alloy sheets were first cut and mounted in resin using a cold mounting procedure. The specimens were then ground with a range of grinding papers (P320, P600, P1200, P2500, and P4000), and polished with a 1 µm diamond suspension. After grinding and polishing, the specimens were etched with Beraha II color etchant and rinsed with laboratory grade alcohol. A Zeiss Axio optical microscope was used to examine the specimens.

### 2.6. Electrochemical Testing

Specimens of both grades of stainless steel were electrochemically tested at three different temperatures: room temperature (22 ± 2 °C), 50 ± 0.5 °C and 90 ± 0.5 °C. A three-electrode corrosion cell was used for the electrochemical tests. Specimens were exposed in the direction normal to the forming direction of the steel sheet. The exposed surface area of the working electrode was 0.785 cm^2^. A saturated Ag/AgCl electrode served as the reference electrode and a graphite electrode was used as the counter electrode. A Gamry Reference 600+ potentiostat/galvanostat was utilized for the measurements, and the accompanying Echem analyst software for analysis of the results. The electrochemical tests were performed after at least 24 h of stabilization at open circuit potential (OCP). Linear polarization measurements were performed at ±20 mV vs. OCP using a scan rate of 0.1 mV/s. Potentiodynamic polarization measurements were carried out immediately after the linear polarization measurements, from −0.2 V vs. OCP towards the anodic direction, at a scan rate of 0.1 mV/s. In order to avoid crevice corrosion, a special double flat inert gasket was used within the electrochemical cell, between the specimen and sample holder. After the measurements, an optical microscope was used at 100× magnification to confirm there was no crevice corrosion.

Data analysis, namely the fitting of Tafel slopes (*β*_a_, *β*_c_), linear polarization resistance (*R*_p_), and determination of electrochemical parameters (corrosion potential *E*_corr_, corrosion current density *j*_corr_), was conducted using Gamry Echem Analyst software. Linear polarization (*R*_p_) was converted to corrosion current density using the Stern-Geary equation (Equation (7)) [29], then the corrosion current density was converted to corrosion rate (*v*_corr_) using Faraday’s law of electrochemical equivalence (Equation (8)) [2]. The following constants were used: atomic weight *a* = 55.1 g/mol and the number of transferred electrons n = 2 for both steel grades, density *ρ* = 8.0 g/cm^3^ for the grade 316L and *ρ* = 7.8 g/cm^3^ for the grade 2507 steel.

(7) $$j_{\mathrm{corr}}=\frac{\beta_{a}\cdot \beta_{c}}{R_{p}\cdot 2.303\cdot \left(\beta_{a}+\beta_{c}\right)}\;\left[A/{\mathrm{cm}}^{2}\right]$$

(8) $$v_{\mathrm{corr}}=\frac{3.27\cdot a\cdot j_{corr}}{\rho \cdot n}\;\left[µm/\mathrm{year}\right]$$

### 2.7. ER Sensor Exposure Tests

**Figure 3:**
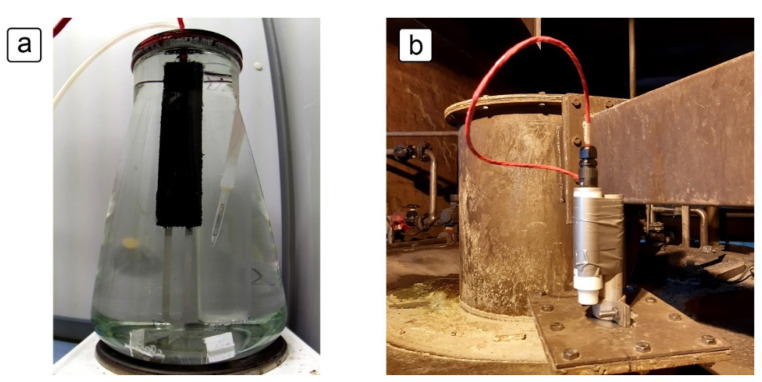
ER sensor exposure setup: (**a**) in the laboratory; (**b**) under industrial conditions.

Multiple ER sensors were tested in order to determine their functionality and accuracy. The tests were done in both controlled laboratory conditions and in fluctuating industrial conditions. Stainless steel grades 316L and 2507 were used in both conditions. Laboratory exposure tests were carried out in a 3000 mL Erlenmeyer flask, as shown in Figure 3a. The temperature was controlled and maintained using a temperature sensor, a proportional integral derivative (PID) controller, and a hot plate below the flask. The mouth of the flask was sealed using custom-made plastic 3D printed lids and polytetrafluoroethylene (PTFE) insulation tape. The ER sensor made of grade 316L steel was exposed to a 10% H_2_SO_4_ solution containing 5000 mg/L NaCl at 65 ± 0.5 °C for 8 days, followed by exposure to the same solution at room temperature for 20 days. The ER sensor made of grade 2507 steel was exposed to a solution of same chemistry, first at 50 ± 0.5 °C for 7 days, then at 65 ± 0.5 °C for 7 days and finally at 90 ± 0.5 °C for 14 days. The 90 °C exposure included a period of 3 days during which the sensor was kept at room temperature (22 ± 2 °C), due to a need to service the heating equipment.

Exposure to industrial conditions was performed in a solution with varying concentrations of sulfuric acid, chloride ions, fluoride ions, ferric ions and arsenic ions. An example of the exposure arrangement is shown in Figure 3b, where a datalogger was strapped to a metal tube with the sensor inside. The cable and tube were long enough to reach the solution inside the tank, such that the sensor was fully immersed in the process solution.

## 3. Results and Discussion

### 3.1. Metallographic Investigation

**Figure 4:**
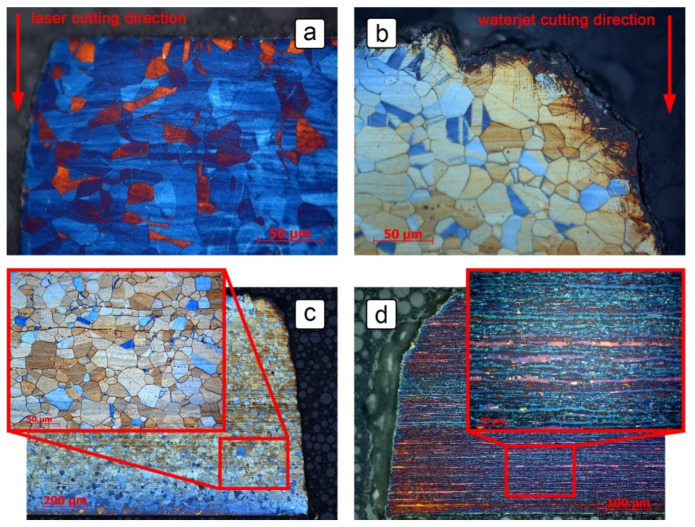
Metallographic images of: (**a**) the angular shape of the laser cut edge in the grade 316L steel sheet; (**b**) strain-induced deformation martensite formed on the edges of the steel sheet cut by abrasive waterjet technique; (**c**) the austenitic microstructure of steel grade 316L with longitudinally oriented inclusions and phases; (**d**) the microstructure of steel grade 2507 with elongated crystal grains in the rolling direction of the steel.

The overall microstructure and the sensor edges from the two cutting procedures were examined in order to determine their effect on the corrosion behavior of the sensor. The changes in microstructure along the edges of the steel sheet can potentially play a major role in service life prediction of the equipment, as there are machine components, such as blades of a mixer, that are exposed to the corrosive environment from all sides during operation. The effect of the laser cutting procedure on grade 316L is shown in Figure 4a, where a cross-section of the edge if the steel sheet following laser cutting is displayed. The edge generally had an angular shape, but it was slightly inclined at an obtuse angle. No indication of recrystallization of the grains was observed near the cut. The grade 2507 steel had similar geometrical and microstructural features along its edges when subjected to laser cutting (not shown in Figure 4).

Figure 4b shows the effect of the abrasive waterjet cutting process on the edge of a grade 316L steel sheet. The edge was heavily damaged at the proximity of the cut on the upper side of the sheet. The cut edge was significantly inclined and visibly rounded. The depth of the damage on the top side ranged from 10 to 30 µm, yet the original sharp edge was removed down to a depth of 150–200 µm. Strain-induced deformation martensite was also formed on the upper corner and upper side of the cut, to a depth of about 50 µm. It was expected that the corrosion resistance of the alloy would be weaker in this area than in the bulk of the material [30]. The grade 2507 showed similar geometrical changes along its edges when subjected to abrasive waterjet cutting, but the depth of the damage was slightly less (10 to 20 µm), and no strain induced martensite was formed. Based on these results, laser cutting was identified as the less intrusive cutting procedure. If not stated otherwise, all specimens used during the research were cut using the laser cutting technique.

The microstructures in the center of the sheet cross-sections are shown in Figure 4c,d, for grades 316L and 2507 respectively. The microstructure of the grade 316L steel consisted of uniform austenitic grains ranging in size between 10 and 40 µm. Strain lines were visible in the rolling direction and were more pronounced and denser in the center of the steel sheet. Some longitudinally oriented inclusions and phases were also present, most likely oxide stringers and δ-ferrite [31]. The microstructure of the grade 2507 was duplex, consisting of austenite in a matrix of ferrite. The grains were fine, no more than a couple of µm in diameter, and heavily extended in the rolling direction.

The microstructures of both grades of stainless steel suggest dissimilar corrosion behavior in the vertical and horizontal directions of the cross-section, as both alloys have visible deformations in the rolling direction. Generally, these deformations tend to reduce the corrosion resistance of the material, so the corrosion rate in the horizontal direction is expected to be higher than that in the vertical direction [12]. To evaluate the corrosion behavior of the stainless steel sheets, ER sensors made of grades 316L and 2507 were exposed to a 10% H_2_SO_4_ solution containing 5000 mg/L NaCl for 28 days at various temperatures. The exposure is described in more detail in Section 3.3., where the assumptions about the dissimilar corrosion rate of the alloys in the directions parallel and normal to the rolling direction were proven.

**Figure 5:**
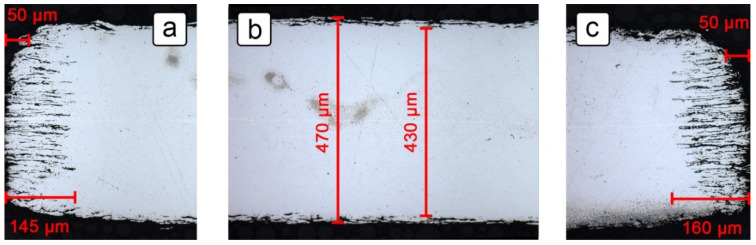
The cross-section reduction of the grade 316L after 28 days of exposure in the 10% H_2_SO_4_ solution containing 5000 mg/L NaCl at 65 °C and at room temperature: (**a**) left edge of a 0.5 mm thick steel sheet; (**b**) middle section of a 0.5 mm thick steel sheet; (**c**) right edge of a 0.5 mm thick steel sheet.

Figure 5 shows parts of the cross-section of the 5 mm wide and 0.5 mm thick 316L stainless steel sheet with visible corrosion damage after 28 days of exposure to the 10% H_2_SO_4_ solution containing 5000 mg/L NaCl at temperatures of up to 65 °C. The thickness of the material in the middle of the cross-section (Figure 5b) ranged from 430 to 470 µm. Since the thickness prior to exposure to the solution was about 500 µm, the corrosion damage on one side of the sheet reached a depth of 15–35 µm. For the purpose of calculating the constant *k* (Equation (4), Figure 2b), a mean value of 25 µm was taken as the *d*_y_ parameter. On the edges of the cross-section, however, determining the dx parameter was more complex. Although the corrosion damage extended up to 160 µm in depth (Figure 5c), this did not reduce the surface area, as modeled in Figure 2b, as bulk steel remained in between the attacked areas. Since maximum corrosion damage is the most critical for the service life of steel equipment, however, the worst-case scenario was considered. A maximum corrosion depth of 160 µm was taken as the *d*_x_ parameter, making the resulting constant *k* equal to 6.

All surfaces and edges of the grade 2507 remained bright and undamaged throughout the 28-day exposure. No corrosion attack was observed along either the free surface, the edges cut by the abrasive waterjet cutting process, the edges cut by the laser cutting process, nor at the liquid-to-air interface.

### 3.2. Electrochemical Tests

**Figure 6:**
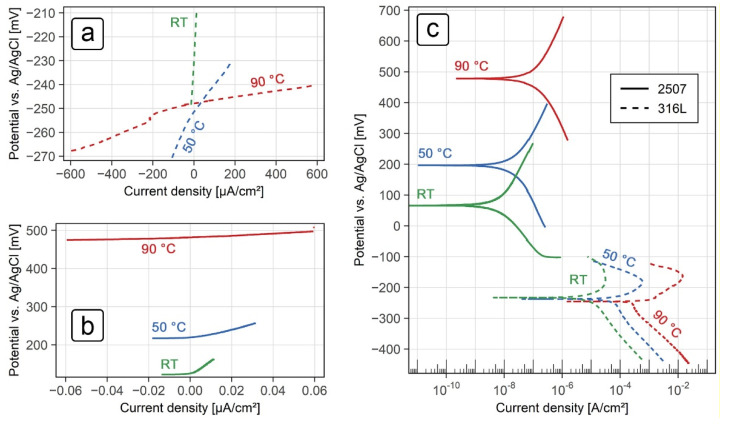
(**a**) Linear polarization curves for the grade 2507 stainless steel in a 10% H_2_SO_4_ solution with 5000 mg/L NaCl at various temperatures; (**b**) linear polarization curves for the grade 316L stainless steel in a 10% H_2_SO_4_ solution with 5000 mg/L NaCl at various temperatures; (**c**) potentiodynamic polarization curves for both grades of stainless steel in a 10% H_2_SO_4_ solution with 5000 mg/L NaCl at various temperatures.

Open circuit potential (OCP), linear polarization and potentiodynamic polarization measurements were performed in order to define the corrosion behavior of the two grades of stainless steel. The tests were performed in a 10% H_2_SO_4_ solution with 5000 mg/L NaCl at room temperature (22 ± 2 °C), 50 ± 0.5 °C and at 90 ± 0.5 °C. The OCP values after 24 h and the electrochemical parameters determined from the measurements for the grade 316L and 2507 steel sheets are shown in Table 2 and Table 3 respectively. Linear polarization curves are shown in Figure 6a (stainless steel grade 316L) and Figure 6b (stainless steel grade 2507), while the potentiodynamic curves for both alloys are shown in Figure 6c.

Polarization resistance values for both grades of stainless steel decreased with an increase in temperature. For the 316L steel, the values of polarization resistance reduced from 1.5 kΩ∙cm^2^ at room temperature to 0.016 kΩ∙cm^2^ at 90 °C (Table 2). Using the Stern-Geary equation, this translated to high corrosion rates, with values of 140 µm/year and 2500 µm/year at room temperature and 90 °C, respectively. Linear polarization values for the steel grade 2507 were about four orders of magnitude higher than the 316L steel, ranging from 3560 kΩ∙cm^2^ at room temperature to about 250 kΩ∙cm^2^ at 90 °C (Table 3). The resulting corrosion rates were much lower compared to the grade 316L, with values of 0.12 µm/year at room temperature and 2.2 µm/year at 90 °C.

The cathodic polarization curves (Figure 6c) for the grade 316L steel showed distinct Tafel regions, whereas the anodic polarization curves for the same material showed an active-to-passive current transition. Evaluation of the corrosion parameters for the grade 316L steel (Table 2) showed that the corrosion potential (*E*_corr_) was around −240 mV vs. Ag/AgCl at all temperatures investigated. Corrosion current densities (*j*_corr_) and the resulting corrosion rates (*v*_corr_), on the other hand, increased significantly with an increase in temperature and indicated active corrosion at all temperatures. The corrosion rates measured were 134 µm/year at room temperature, 795 µm/year at 50 °C and 2790 µm/year at 90 °C.

Potentiodynamic polarization curves for the grade 2507 steel (Figure 6c) showed well-defined anodic and cathodic Tafel regions at all temperatures investigated. Both the corrosion potential (*E*_corr_) and the corrosion current density (*j*_corr_) increased with an increase in temperature (Table 3). *E*_corr_ values at room temperature, 50 °C and 90 °C were approximately 70 mV, 200 mV and 480 mV respectively, while the corrosion rates, calculated from the corrosion current densities, were approximately 0.09 µm/year, 0.32 µm/year and 1.6 µm/year, respectively.

Overall, comparable results were seen between the two electrochemical techniques in both grades of stainless steel. These results are also consistent with the corrosion behavior observed, given that no noticeable corrosion damage was seen in the grade 2507 steel after exposure to the test solution at various temperatures for 28 days, whereas excessive corrosion damage was seen in grade 316L steel after exposure to the same conditions.

### 3.3. ER Sensor Results in the Laboratory

**Figure 7:**
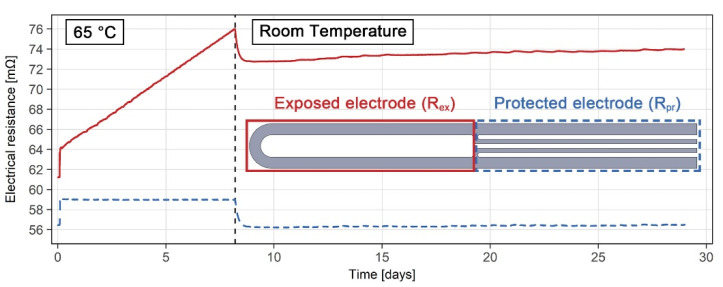
Electrical resistance results for the grade 316L ER sensor exposed to a 10% H_2_SO_4_ solution with 5000 mg/L NaCl at 65 °C and room temperature.

In order to test the validity and accuracy of the ER sensor measurements, and the durability of the various sensor components, an ER sensor made of grade 316L steel was exposed to the 10% H_2_SO_4_ solution with 5000 mg/L NaCl for 28 days, at 65 °C and at room temperature. Results showing the electrical resistance of the exposed working electrode (*R*_ex_) and the protected electrode (*R*_pr_) are shown in Figure 7. The initial electrical resistances of the exposed and protected part of the 316L probe at room temperature were measured as 61.23 mΩ and 56.74 mΩ, respectively. During the first 8 days of exposure, the temperature was maintained at 65 °C. The electrical resistance of the protected electrode was very stable during this period, maintaining a value of 59.0 mΩ. The electrical resistance of the working electrode, on the other hand, changed significantly, increasing from 64.1 mΩ to 76.0 mΩ during the same period. The necessity for temperature compensation was clearly shown in the second part of the exposure period, when the sensor was exposed to changes in room temperature. Immediately following the end of the 65 °C exposure period (indicated by the dashed vertical line in Figure 7), a drop in the electrical resistance of several mΩ could be observed in both the exposed and the protected electrodes. Additionally, as the exposure continued, daily variations and a slow general increase in electrical resistance were detected in both the exposed and protected electrodes. Both such effects were compensated for when analyzing the results.

**Figure 8:**
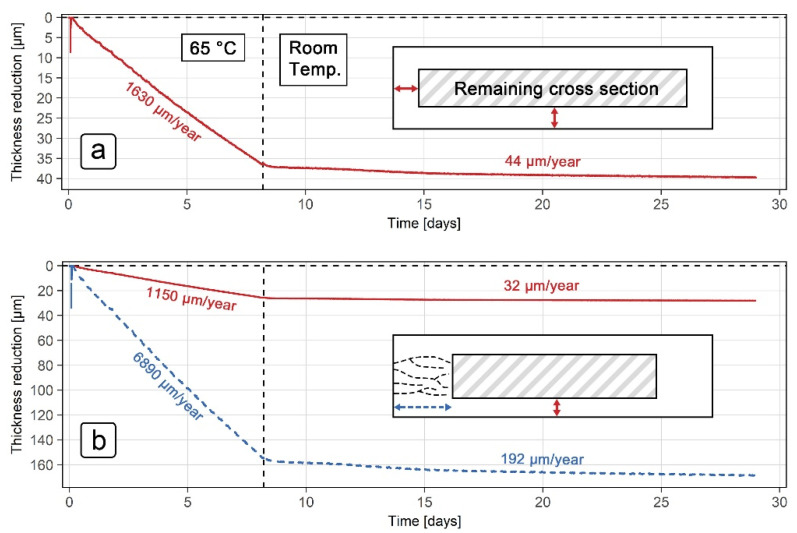
ER thickness reduction results for grade 316L steel in the 10% H_2_SO_4_ solution with 5000 mg/L NaCl: (**a**) model using the same cross-section reduction in vertical and horizontal direction; (**b**) model using the maximum reduction in cross-section in the horizontal direction.

The thickness reduction results for the grade 316L ER probe in a 10% H_2_SO_4_ solution with 5000 mg/L NaCl are shown in Figure 8. The results are based on the same ER sensor measurement data as demonstrated in Figure 7, but presented using two different thickness reduction models. In the first model (Figure 8a), it was assumed that the same thickness reduction occurred in the normal and transversal directions of the cross-section (*k* = 1 in Equations (5) and (6)), while the second model (Figure 8b) uses the maximum thickness reduction in the transversal direction of the cross-section (k = 6 in Equations (5) and (6)), as described in Section 2.2 and Section 3.1. A schematic representation of the cross-section was also included in both models, with colored and dashed arrows representing the different thickness reductions. The vertical dashed line at day 8 shows the transition point between exposure to 65 °C and room temperature conditions.

When interpreting the ER sensor measurements according to the first model, where the thickness reduction in both directions of the cross-section is the same (Figure 8a), the corrosion rate is shown to be 1630 µm/year at 65 °C and 44 µm/year at room temperature. The results for the 65 °C exposure were in line with those obtained using the electrochemical techniques (870 µm/year at 50 °C and 2650 µm/year at 90 °C, as shown in Table 4), while the corrosion rate at room temperature was about 3 times lower compared to that found by the electrochemical results (around 140 µm/year, Table 4). When comparing the modeled thickness reduction with the corrosion damage observed at the end of the exposure, however, the first model showed additional discrepancies in the results obtained. Postexposure examination revealed corrosion damage to be around 25 µm in the normal direction and up to 160 µm in the transverse direction, whereas interpretation of the ER results (Figure 8a) showed that the thickness reduction at the end of the exposure should have been around 40 µm in both directions of the cross-section.

The second model, shown in Figure 8b, corrects this disparity by separating the reduction in thickness into normal and transverse directions. According to the improved interpretation of the ER results, the reduction in thickness at the end of the exposure was 27 µm in the normal direction and 165 µm in the transverse direction. The corrosion rates in the second model (Figure 8b) were also modified accordingly in both directions. In the normal direction, the values for the corrosion rate decreased slightly compared to the first model, specifically 1150 µm/year at 65 °C and 32 µm/year at room temperature (solid line, Figure 8b), whereas the corrosion rates in the transverse direction increased significantly (dashed line, Figure 8b), with new values of 6890 µm/year and 192 µm/year at 65 °C and room temperature, respectively. Although the corrosion rates in the normal direction remained comparable to corrosion rates obtained using the electrochemical techniques (Table 4), corrosion rates in the transverse direction increased by a factor of 6, showing the importance of taking into account the microstructure when evaluating corrosion rates from ER measurements.

The measured corrosion rates can also be used to approximate probe lifetimes. At room temperature, the 316L probe would last for roughly 16 years, while at 65 °C, the same probe would last for only 5 months. Scaling these results to an acid tank with a 5 mm thick wall, the service life for maintaining integrity would be about 150 years at room temperature and 4 years at 65 °C. Reduced load capacity of the tank would become a problem much before this service life is achieved, however.

**Figure 9:**
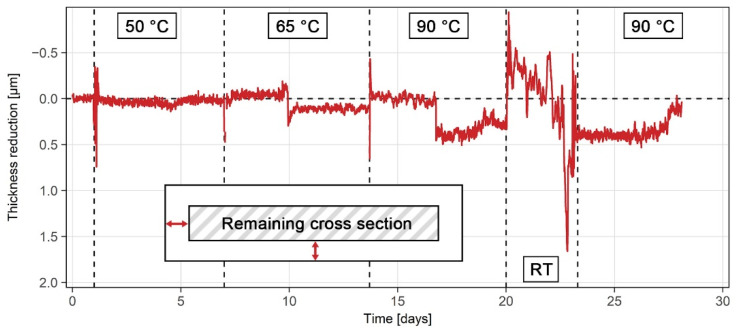
ER thickness reduction results for the grade 2507 steel during 28 days of exposure to a 10% H_2_SO_4_ solution with 5000 mg/L NaCl at various temperatures.

In addition to exposure of the grade-316L sensor, the ER sensor made of grade 2507 steel was similarly exposed to the 10% H_2_SO_4_ solution with 5000 mg/L NaCl for 28 days. Since the postexposure examination did not show any notable damage to the grade 2507, the *k* value in Equation (4) was assumed to be 1. The initial electrical resistances of the exposed and protected part of the 2507 probe at room temperature prior to the exposure were measured as 68.36 mΩ and 63.00 mΩ, respectively. The thickness reduction results for the steel grade 2507 are presented in Figure 9. The results were consistent both with observations of corrosion behavior and the electrochemical measurements. Minimal corrosion was recorded at all temperatures (Table 3 and Table 4), which also suggests corrosion would not impact the service life of equipment made from such steel. Only some discrete jumps, up to 0.5 µm around the baseline, were detected at 10, 14, 17, 20 and 24 days of exposure (Figure 9). These perturbations were present due to external experimental factors, such as physically moving the sensor or the measuring device, changing the temperature around either the sensor or the measuring device, turning the heating system on and off, and refilling the solution due to evaporation. Once enough corrosion occurs (equating to a reduction in thickness of at least 2–3 µm), the impact of these external factors on determining accurate corrosion rates significantly reduces.

Although the corrosion rates obtained from ER sensors in the normal direction were slightly lower compared to the electrochemical measurements (Table 4), they were generally within the same order of magnitude for both steel grades and at all evaluated temperatures. This can be explained by the fact that the stainless steel sheets were exposed in the normal direction only during electrochemical testing, and because the use of polarization modifies the surface as compared to the surface in an equilibrium system. Since the microstructure in the transverse direction evidently contributed to the corrosion losses, simultaneous exposure in both directions during the ER sensor tests likely changed the electrochemical system and subsequently influenced the corrosion rates measured.

### 3.4. ER Sensor Results from Exposure under Industrial Conditions

**Figure 10:**
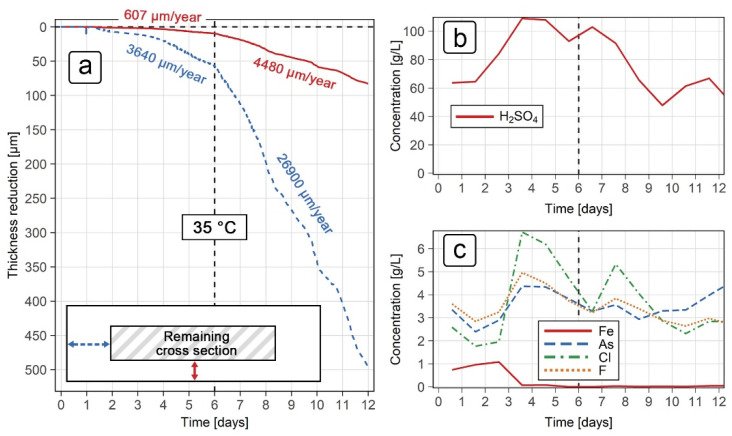
Exposure of grade 316L ER sensor under industrial conditions: (**a**) thickness reduction results; (**b**) concentration of sulfuric acid; (**c**) concentration of the various ions.

**Figure 11:**
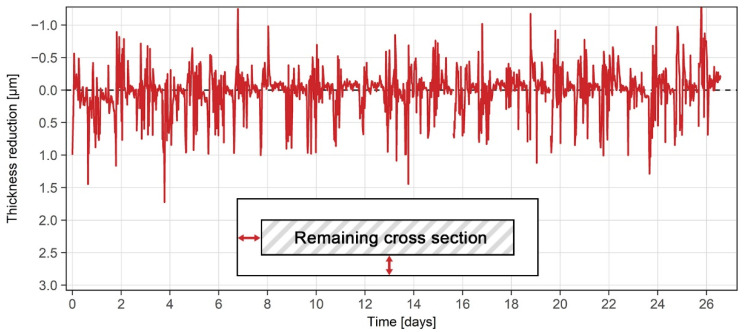
Reduction in thickness of the grade 2507 ER sensor under industrial conditions.

In order to test the behavior of the ER sensor in real, uncontrolled conditions, both steel grades were exposed to solution under industrial conditions (Figure 1a and Figure 3b). The reduction in thickness for the grades 316L and 2507 is shown in Figure 10a and Figure 11, respectively. When interpreting the thickness reduction from the resistance measurements, the same *k* values were taken as for the laboratory exposure for both grades of steel (*k* = 6 for grade 316L and *k* = 1 for grade 2507). The varying composition of the solution to which the grade 316L sensor was exposed is also shown in Figure 10b (H_2_SO_4_ concentration) and Figure 10c (various ion concentrations). The grade 2507 was exposed to similar conditions at a later time.

The results for the grade 316L sensor (Figure 10a) show two distinct regimes with relatively stable corrosion rates, which occurred during the first and last 6 days of exposure. In the first 6 days of exposure, the corrosion rates were measured to be around 600 µm/year in the normal direction, and around 3640 µm/year in the transverse direction. Both corrosion rates increased significantly after day 6, with average values of 4480 µm/year in the normal and 26,900 µm/year in the transverse direction. Such high corrosion rates indicated that, even in the relatively short 6-day exposure, severe corrosion damage was expected. The expected lifetime of a grade 316L ER probe in such conditions would be 1 month at most, provided no mechanical damage or localized corrosion occurs. Additionally, a 5 mm thick acid tank made from this steel grade would keep its integrity for less than 1 year. Throughout the exposure, the temperature remained relatively stable at 35 °C, while the concentrations of both sulfuric acid (Figure 10b) and the various ions (Figure 10c) changed over time. The results show that, in the second half of the exposure, the concentrations of sulfuric acid, chloride ions and fluoride ions decreased compared to the first 6 days of the exposure, indicating that such conditions were beneficial for corrosion in the grade 316L steel. The sulfuric acid and the chloride ion concentrations dropped by approximately 50% (from 110 to 60 g/L, and from 6 to 3 g/L, respectively), while the fluoride ion concentration lowered from 5 to 3 g/L. The concentration of arsenic ions remained relatively stable, hovering between 3 and 4 g/L, while the iron concentration was only significant during the first 3 days of exposure (1 g/L). The presence of fluoride and arsenic ions was likely the reason for the significantly higher corrosion rate of the material under industrial conditions compared to the laboratory tests.

Corrosion monitoring stopped shortly after 12 days of exposure, as the sensor indicated that the electrical current flow over the U-shaped working electrode was interrupted. This was likely due to excessive damage in the transverse direction and mechanical wear due to liquid current flowing through the industrial process tanks. Postexposure examination revealed that the sensor was severely corroded, with parts of the working electrode missing, most likely dissolved in the acidic solution. The sensor was taken out of the tank about two weeks after the monitoring stopped; it showed excessive damage beyond what was measured towards the end of the exposure (Figure 10a). An accurate comparison of the corrosion damage with the thickness measurements was not possible.

The reduction in thickness of grade 2507 steel indicated that no notable corrosion processes took place during the first 26 days of exposure, as shown in Figure 11. The noise levels of ±1 µm around the baseline were likely due to the turbulent nature of the acidic solution inside the industrial process tank. After 26 days of exposure, the sensor was removed from the tank for maintenance purposes. After some solution deposits on the surface were cleaned, the sensor was examined and showed no corrosion damage. Since the grade 2507 duplex stainless steel was designed to withstand the sulfuric acid environments, the corrosion results measured and observed were as expected. The exposure of the grade 2507 sensor will likely continue over the coming years, as a minimum reduction of 2 µm in thickness will be needed to obtain accurate corrosion rates. Such an extended period of exposure will also fully test the integrity and durability of the sensor and reveal any unpredicted weaknesses in the design.

## 4. Conclusions

This study described the development of an electrical resistance sensor for monitoring the corrosion of stainless steel in the hydrometallurgical industry. The sensors, made from two grades of stainless steel, were designed for harsh corrosive environments involving sulfuric acid and high concentrations of chlorides at temperatures of up to 90 °C. The sensor design, the corrosion behavior of the materials, the corrosion monitoring capability, and evaluation of the results measured both in the laboratory and under industrial process conditions were presented. The following conclusions were made.

1. The important outcome of the study was the demonstration of performance of the ER sensor for corrosion monitoring in the hydrometallurgical industrial environment since conventional electrochemical techniques cannot be successfully applied during operation.
2. The design enabled the sensors to withstand aggressive sulfuric acid environments and enabled continuous monitoring of the reduction in material thickness during the hydrometallurgical processes under varying operating conditions.
3. Electrical resistance sensors were made of two grades of stainless steel, both of which are used in industrial process applications as structural materials in pipes and tanks. The sensor development took the microstructure of the stainless steel, which played a large role in determining the corrosion rate, into account. This showed the importance of using an appropriate stainless steel grade and cutting process in sensor production.
4. The sensors were made from electrically insulating protective materials resistant to extremely acidic environments and high temperatures to which they would be exposed and were built to be disposable. Long-term industrial exposure is still needed to fully test the integrity and durability of the sensor and its components.
5. In the direction normal to the rolling direction, the corrosion rates obtained from continuously monitoring the ER sensors were generally in agreement with the corrosion rates obtained using the electrochemical measurements. In the transverse direction, the ER sensor showed corrosion rates an order of magnitude higher in comparison to the normal direction.
6. Both the ER sensor measurements and the electrochemical tests showed significantly higher corrosion rates in the grade 316L stainless steel compared to the grade 2507 steel. The corrosion rates measured for the grade 316L steel at 90 °C were in excess of 2 mm/year, while the corrosion rates for the grade 2507 steel were about 3 orders of magnitude lower at the same temperature. This reflects that the two types of sensors serve for environments with more or less harsh conditions, and that the sensors of grade 316L may be better suited for milder industrial process conditions.

## Figures and Tables

**Table 1:** Composition and pitting resistance equivalent numbers (PRENs) of the 316L and 2507 grade stainless steels.

AISI.	316L	2507
EN	1.4404	1.4410
PREN	24	42
Cr (%)	17.1	25.9
Ni (%)	10.0	7.24
Mo (%)	2.01	3.75
N (%)	0.04	0.35
C (%)	0.030	0.027

**Table 2:** Electrochemical parameters (*E*_corr_ and OCP (mV), *β*_x_ (mV), *R*_p_ (kΩ∙cm^2^), *j*_corr_ (µA/cm^2^) and *v*_corr_ (µm/year)) extracted from OCP, linear polarization and potentiodynamic polarization curves for the grade 316L stainless steel exposed to the 10% H_2_SO_4_ solution with 5000 mg/L NaCl at various temperatures.

Temp.	Linear Polarization			Potentiodynamic Polarization					OCP at 24 h
	*R*_p_	*j*_corr_	*v*_corr_	*β*_a_	*β*_c_	*j*_corr_	*v*_corr_	*E*_corr_	
RT	1.46	12.5	140	61	134	11.9	134	−233	−235
50 °C	0.147	83.3	938	32	216	70.6	795	−238	−250
90 °C	0.016	222	2500	8.8	158	248	2790	−246	−250

**Table 3:** Electrochemical parameters (*E*_corr_ and OCP (mV), *β*_x_ (mV), *R*_p_ (kΩ cm^2^), *j*_corr_ (µA/cm^2^) and *v*_corr_ (µm/year)) extracted from OCP, potentiodynamic polarization and linear polarization curves for grade 2507 stainless steel exposed to a 10% H_2_SO_4_ solution with 5000 mg/L NaCl at various temperatures.

Temp.	Linear Polarization			Potentiodynamic Polarization					OCP at 24 h
	*R*_p_	*j*_corr_	*v*_corr_	*β*_a_	*β*_c_	*j*_corr_	*v*_corr_	*E*_corr_	
RT	3560	0.01	0.12	221	138	0.008	0.09	66	133
50 °C	1260	0.04	0.46	229	237	0.028	0.32	196	202
90 °C	252	0.19	2.23	272	191	0.135	1.56	478	482

**Table 4:** Comparison of corrosion rates (µm/year) in the direction normal to the rolling direction for the grade 316L and grade 2507 steel, obtained with electrochemical techniques and ER sensors.

Steel	Temperature	Electrochemical Techniques	ER Sensor
316L	RT	140	32
	50 °C	870	-
	65 °C	-	1150
	90 °C	2650	-
2507	RT	0.1	-
	50 °C	0.4	<1 *
	65 °C	-	<1 *
	90 °C	2.0	<1 *

* no detected thickness reduction during exposure.

## Data Availability

The data that support the findings of this study are available on reasonable request from the corresponding author.
